# A cross-sectional study evidences regulations of leukocytes in the colostrum of mothers with obesity

**DOI:** 10.1186/s12916-022-02575-y

**Published:** 2022-11-01

**Authors:** Raúl Piñeiro-Salvador, Eduardo Vazquez-Garza, José Antonio Cruz-Cardenas, Cuauhtémoc Licona-Cassani, Gerardo García-Rivas, Jorge Moreno-Vásquez, Mario René Alcorta-García, Victor Javier Lara-Diaz, Marion E. G. Brunck

**Affiliations:** 1 Tecnologico de Monterrey, Escuela de Ingeniería y Ciencias, Av. Eugenio Garza Sada 2501 Sur, Tecnologico, 64849 Monterrey, Nuevo León Mexico; 2 Tecnologico de Monterrey, Escuela de Medicina y Ciencias de la Salud, Ave. Morones Prieto 3000 Poniente, Col. Doctores, 64710 Monterrey, Nuevo León Mexico; 3 The Institute for Obesity Research, Tecnologico de Monterrey, Av. Eugenio Garza Sada 2501 Sur, Tecnologico, 64849 Monterrey, Nuevo León Mexico; 4 Hospital Regional Materno-Infantil, SSNL, OPD, Ciudad Guadalupe, Nuevo León Mexico

**Keywords:** Colostrum, Leukocytes, Obesity, Flow cytometry

## Abstract

**Background:**

Breastmilk is a dynamic fluid whose initial function is to provide the most adapted nutrition to the neonate. Additional attributes have been recently ascribed to breastmilk, with the evidence of a specific microbiota and the presence of various components of the immune system, such as cytokines and leukocytes. The composition of breastmilk varies through time, according to the health status of mother and child, and altogether contributes to the future health of the infant. Obesity is a rising condition worldwide that creates a state of systemic, chronic inflammation including leukocytosis. Here, we asked whether colostrum, the milk produced within the first 48 h post-partum, would contain a distinct leukocyte composition depending on the body mass index (BMI) of the mother.

**Methods:**

We collected peripheral blood and colostrum paired samples from obese (BMI > 30) and lean (BMI < 25) mothers within 48 h post-partum and applied a panel of 6 antibodies plus a viability marker to characterize 10 major leukocyte subpopulations using flow cytometry.

**Results:**

The size, internal complexity, and surface expression of CD45 and CD16 of multiple leukocyte subpopulations were selectively regulated between blood and colostrum irrespective of the study groups, suggesting a generalized cell-specific phenotype alteration. In obesity, the colostrum B lymphocyte compartment was significantly reduced, and CD16^+^ blood monocytes had an increased CD16 expression compared to the lean group.

**Conclusions:**

This is the first characterization of major leukocyte subsets in colostrum of mothers suffering from obesity and the first report of colostrum leukocyte subpopulations in Latin America. We evidence various significant alterations of most leukocyte populations between blood and colostrum and demonstrate a decreased colostrum B lymphocyte fraction in obesity. This pioneering study is a stepping stone to further investigate active immunity in human breastmilk.

**Supplementary Information:**

The online version contains supplementary material available at 10.1186/s12916-022-02575-y.

## Background

Human breastmilk has been historically regarded as a source of nutrition for infants. Recent studies have evidenced that breastmilk is a complex and dynamic tissue that provides newborns with components involved with functions beyond nutrition, such as the breastmilk microbiota and mother-derived cytokines and leukocytes [1]. The production and composition of breastmilk are partially modulated by external parameters such as the mother’s diet, stress levels, or health status [2–4].

Obesity is an expanding public health problem worldwide that can be regarded as a state of low-grade systemic inflammation where larger adipocytes secrete proinflammatory mediators and recruit leukocytes [5, 6]. Individuals suffering from obesity exhibit altered peripheral blood cell counts with increased risks of leukocytosis, and modulations in the phenotypes of lymphocyte subpopulations [7, 8]. Obesity directly hampers breastfeeding by various mechanisms, including delayed lactogenesis, decreased milk supply, and issues in adequately positioning the infant, all of which have been previously described [9–11]. In addition, obesity impacts micro- and macronutrient breastmilk composition and selectively regulates its abundance in soluble immune components. Higher levels of immunoglobulin A (IgA) and secretory IgA (sIgA) concentrations were found in obese serum and colostrum, respectively, while IgG and IgM concentrations were unaffected by maternal body mass index (BMI) [12]. The relevance of increased sIgA in obese colostrum remains to be elucidated. It may be a consequence of the observed disruption of the microbiota in these samples. In Mexico, the colostrum from obese mothers overall contains a microbiota with more bacterial species (increased richness) and more diversity between species abundances (decreased evenness) compared to colostrum microbiota from lean mothers [13, 14]. Obese colostrum microbiota also include more potentially pathogenic bacteria genus such as *Staphylococcus* [14]. This may also partly explain the regulation of immune soluble factors described in obese breastmilk, including decreased TGF-β and sCD14, while IL-1 β, IL-6, IL-8, IL-10, and TNF- α concentrations were not significantly altered [15–17].

While historical empirical observations have associated breastfeeding with a moderately decreased risk of suffering from obesity later in life, in these studies, the weight status of breastfeeding mothers was not investigated and may be a confounding factor skewing conclusions [18, 19]. Indeed, overall maternal obesity is associated with multiple immune-mediated negative outcomes for infants, including neurodevelopmental disorders and increased morbidity [20–22]. To date, these outcomes have been discussed as consequences of epigenetic regulations and gut microbiota alterations in early life [22]. Recent evidence suggests breastmilk-transferred immune factors may impact infant health as the transfer of immune factors through breastmilk may also promote the development of autoimmune conditions [23, 24]. However, the consequences of obesity on the majority of breastmilk leukocyte populations have not been reported to date [25].

As alterations in breastmilk immune components could impact infants’ future health, we sought to investigate the consequence of obesity on breastmilk leukocytes in a cross-section observational study [25]. The primary objective of this study was to explore possible variations in leukocyte proportions in the colostrum of mothers suffering from obesity. A secondary objective of this work was to compare the proportions and characteristics of leukocytes depending on the tissue of origin: colostrum or peripheral blood.

## Methods

### Study design and participants

We conducted a cross-sectional study of leukocyte subpopulations in blood and colostrum of mothers with BMI <25 and BMI >30. This study was approved by the Ethics Committee of the Hospital Regional Materno Infantil, Servicios de Salud de Nuevo León, Mexico, and the Institutional Review Board at Escuela de Medicina y Ciencias de la Salud, TecSalud, in Monterrey, Mexico, with the ID CarMicrobioLHum-2018. All samples were collected and used following signed informed consent and anonymization, between October 2020 and March 2021 at the Hospital Regional Materno Infantil, in Nuevo León, Mexico. Briefly, adult mothers between 18 and 34 years of age were invited to participate during the first obstetric consultation occurring during the first trimester of gestation. Participants were allocated to the obese cohort (BMI >30) or lean cohort (BMI <25), according to declared pre-pregnancy weight during the first visit and in accordance with the World Health Organization classification guidelines [26]. Eligibility to participate in the study was determined based on (1) mother’s age between 18 and 34 years, (2) adequate prenatal visits without any adverse event during pregnancy, (3) pre-pregnancy BMI <25 or >30, (4) term infant, and (5) willingness to participate. Exclusion criteria included (1) having received antibiotics anytime during the 3-month period before birth, or having received a prolonged antibiotic treatment (>3 months) anytime during pregnancy; (2) having received immunosuppressive doses of steroids during pregnancy; (3) previous monoclonal antibody treatment; (4) history of chronic disease (outside of obesity); (5) suffering from any dietary disease; (6) episodes of diarrhea during the last 2 weeks of pregnancy; (7) history of surgery within 12 months prior to pregnancy; and (8) history of antineoplastic treatment. Elimination criteria included (1) having received antibiotics for >24 h post-birth, (2) necessity of intensive care unit admission of the neonate, and (3) any additional cause impeding sample collection. Oxytocin was not used during the first stages of labor. However, oxytocin was prescribed in 34 of 41 subjects (84%), during the first 8 h after delivery, as per international recommendations [27].

Regarding the variables of the study, the main independent variable and hypothesized predictor was the BMI, calculated from self-reported pre-pregnancy weight and size. Additional variables collected or measured in this work included participant’s age, primiparity (yes/no), infant gender, gestational age at birth, type of delivery (vaginal/C-section), weight of infant at birth, volume of colostrum obtained, and frequency of leukocyte subpopulations in blood and colostrum samples. Additional details are included in the study’s STROBE statement (Sup. Table 1) [26, 28–31].

Participants provided blood and colostrum samples on a single occasion, within 2 days of giving birth. Briefly, 3–4 ml of peripheral blood was collected in K2-EDTA vacutainers and placed on ice until processing. Following washing of the breast and nipple area using soap and water, 1–3 ml of colostrum per donor was obtained through pump-assisted milk extraction and immediately stored on ice. All samples were processed and acquired on the flow cytometer within 3 h of collection.

### Leukocyte enrichment from colostrum

Around 1 ml of colostrum was processed for cell enrichment prior to staining for flow cytometry. Briefly, samples were centrifuged at 400 g for 15 min at 4°C. The supernatant was discarded, and the cell pellet was washed twice with PBS/2% FBS. Cells were counted using trypan blue for viability assessment and aliquoted for flow cytometry staining.

### Colostrum-enriched cell staining

Depending on availability, between 200,000 and 1 × 10^6^ cells were used for staining, and the same number of cells per sample was kept as unstained control. The same antibody lots were used to stain both types of tissues and antibody titration optimizations were performed for each tissue type to optimize resolution of fluorescence intensity over background. Cells were resuspended in the antibody master mix, which consisted of 2.5 μL mouse anti-human CD2-APC (BD® cat. 560642), 5 μL mouse anti-human CD16-APC-H7 (BD® cat. 560195), 5 μL mouse anti-human CD19-V450 (BD® cat. 560353), 2.5 μL mouse anti-human CD36-PE (BD® cat. 555455), 5 μL mouse anti-human CD45-V500 (BD® cat. 560777), and 1.25 μL rat anti-human CD294-Alexa Fluor 647 (BD® cat. 558042), in a final 100-μL staining volume with PBS + 2% FBS per 10^6^ cells. Samples were then incubated for 30 min on ice in the dark, then washed and resuspended in PBS/2% FBS. Ten min before acquisition, propidium iodide (BD® cat. 556463) was added to the tube as per the manufacturer’s recommendations.

### Peripheral blood staining

Fifty microliters of anticoagulated peripheral blood was stained using 2.5 μL CD2-APC, 1.25 μL CD16-APC-H7, 5μL CD19-V450, 2.5 μL CD36-PE, 1.25 μL CD45-V500, and 1.25 μL CD294-Alexa Fluor 647, in a final 100-μL staining volume with PBS + 2% FBS for 30 minutes on ice, in the dark. Samples were then subjected to erythrocyte lysis using BD® Pharm Lyse solution (BD® cat. 555899) as per the manufacturer’s instructions. Ten minutes before acquisition propidium iodide was added to tubes as per the manufacturer’s recommendations.

### Flow cytometry data acquisition

Samples were analyzed on a BD® FACSCelesta flow cytometer fitted with 405-nm, 488-nm, and 633-nm lasers and operated through the BD® FACSDiva software v.8. Cytometer settings were checked prior to all acquisition using CS&T beads (BD® cat. 642412) according to manufacturer’s instructions. Compensation controls were prepared using compensation beads (anti-mouse Ig, K Neg Control compensation, BD® cat. 552843) following the manufacturer’s recommendations. At least 30,000 uncompensated events were recorded from every sample, with the forward scatter (FSC) event threshold adjusted to 35,000 for peripheral blood and 28,000 for colostrum samples.

### Flow cytometry data analysis

Cytometry data were analyzed using FlowJo software v.10 (Treestar LLC). Automatic compensation was performed prior to analysis, with a compensation matrix generated at each acquisition. A strict quality control workflow was established to ensure the exclusion of suboptimal quality samples that may artificially skew the final analysis (Fig. S1) [32]. Briefly, samples had to exhibit a stable flow stream (Side Scatter (SSC) vs. time), debris was excluded on SSC/FSC plot, sample viability >85% from the singlet gate, and >10,000 leukocytes (CD45^+^ cells) acquired, to be included in the final analysis and comparisons [33]. The gating strategy applied to discriminate the leukocyte populations has been previously described and is summarized in Table 1 [31, 34]. Fluorescence minus one (FMO) controls of colostrum and blood samples were used to adjust gates, which were then applied to all samples.

**Table 1:** Flow cytometry qualitative thresholds considered to identify the investigated leukocyte populations

Cell type	Phenotype
**Granulocytes**	
Neutrophils	SSC^bright^, CD45^+^, CD16^+^
Eosinophils	SSC^bright^, CD45^+^, CD16^-^, CD2 / CD294^+^
Basophils	SSC^int^, CD45^+^, CD16^-^, CD2 / CD294^+^
**Lymphoid lineage cells**	
Noncytotoxic T lymphocytes	SSC^dim^, CD45^+^, CD16^-^, CD2 / CD294^+^
Cytotoxic T/NK lymphocytes	SSC^dim^, CD45^+^, CD16^+^, CD2 / CD294^+^
B lymphocytes	SSC^dim^, CD45^+^, CD16^-^, CD2 / CD294^-^, CD19^+^
**Monocytes**	
CD16- (classical) monocytes	SSC^int^, CD45^+^, CD16^-^, CD2 / CD294^-^, CD19^-^, CD36^+^ CD16^-^
CD16+ (non-classical) monocytes	SSC^int^, CD45^+^, CD16^+^, CD2 / CD294^-^, CD19^-^, CD36^+^, CD16^+^
**Precursors/Immature cells**	
Myeloid precursors	SSC^dim^, CD45^+^, CD19^-^, CD2/ CD294^-^
Immature granulocytes	SSC^bright^, CD45^+^, CD16^-^, CD2 / CD294^-^

### Statistical analysis

Proportions of leukocyte subsets were calculated as % of CD45^+^ live cells per sample. Shapiro-Wilk tests were used to investigate data normality with *α*=0.05. Wilcoxon matched-pairs tests were used to compare intra-individual leukocyte proportions in paired blood-colostrum samples. Mann-Whitney *U* tests were used to compare leukocyte % and median fluorescence intensity (MFI) of surface markers in colostrum between study groups. Student *t*-tests were used to compare leukocyte proportions in blood samples. All statistical analyses were performed using GraphPad Prism v. 9, or SPSS® v. 26, IBM Corporation, Armonk, NY, USA. Graphs are showing discrete data and mean with SD and *p* values in the APA format.

## Results

A total of 41 participants were enrolled in this study, with 21 mothers allocated to the lean BMI group and 20 mothers allocated to the obese BMI group. Post-acquisition quality filters on flow cytometry preliminary data restricted the final analysis to 21 blood samples and 17 colostrum samples in the lean BMI group, and 20 blood samples and 11 colostrum samples in the obese BMI group (Sup. Fig. 1). An overall summary of the clinical variables of each study group is presented in Table 2. As per the study design, the average BMI was significantly larger in the obese mothers’ cohort (34.9 vs. 22.8 in the lean cohort, *p*<0.001). There was no difference in maternal age, gestational age at delivery, mode of delivery (C-section or vaginal), gender of infant, weight of infant at birth, or primiparity between groups, and post-quality filter sample exclusion did not unearth additional difference between groups (Sup. Table 2 and 3). The average volume of collected colostrum was significantly smaller in the obese mothers’ cohort (1.42 ml vs 2.11 ml).

**Table 2:** Summary of demographic and clinical parameters of study participants included in the blood analysis

	Cohort		*p*
Variable	Lean	Obese	
N total	21	20	
Maternal age (years), median (IQR)	23 (22-26)	26 (21-29)	0.82
Maternal BMI (kg/m^2^), median (IQR)	22.6 (21.1-23.9)	34.5 (33.7-35.4)	**<0.001**
Primiparous, N (%)	6 (29)	6 (30)	0.09
Infant gender, N females (% total)	9 (43)	8 (40)	1
Gestational age (weeks), median (IQR)	39 (38-40)	39 (38-40)	0.09
Delivery type, N V (%)	15 (71)	13 (65)	0.74
Infant birth weight (g), median (IQR)	3340 (3000-3850)	3412 (3036-3619)	0.46
Volume of colostrum obtained (mL), median (IQR)	2.0 (1.5-2.5)	1.5 (1.0-1.7)	**0.008**

*N* number of events, *BMI* body mass index, *V* vaginal births, *IQR* interquartile range. Statistically significant *p* values for the calculated differences are depicted in bold type. Continuous data were analyzed with Mann-Whitney’s *U* test, and proportions were compared with Fisher’s exact test

### No variation is observed in blood leukocyte proportions between cohorts

**Fig. 1:**
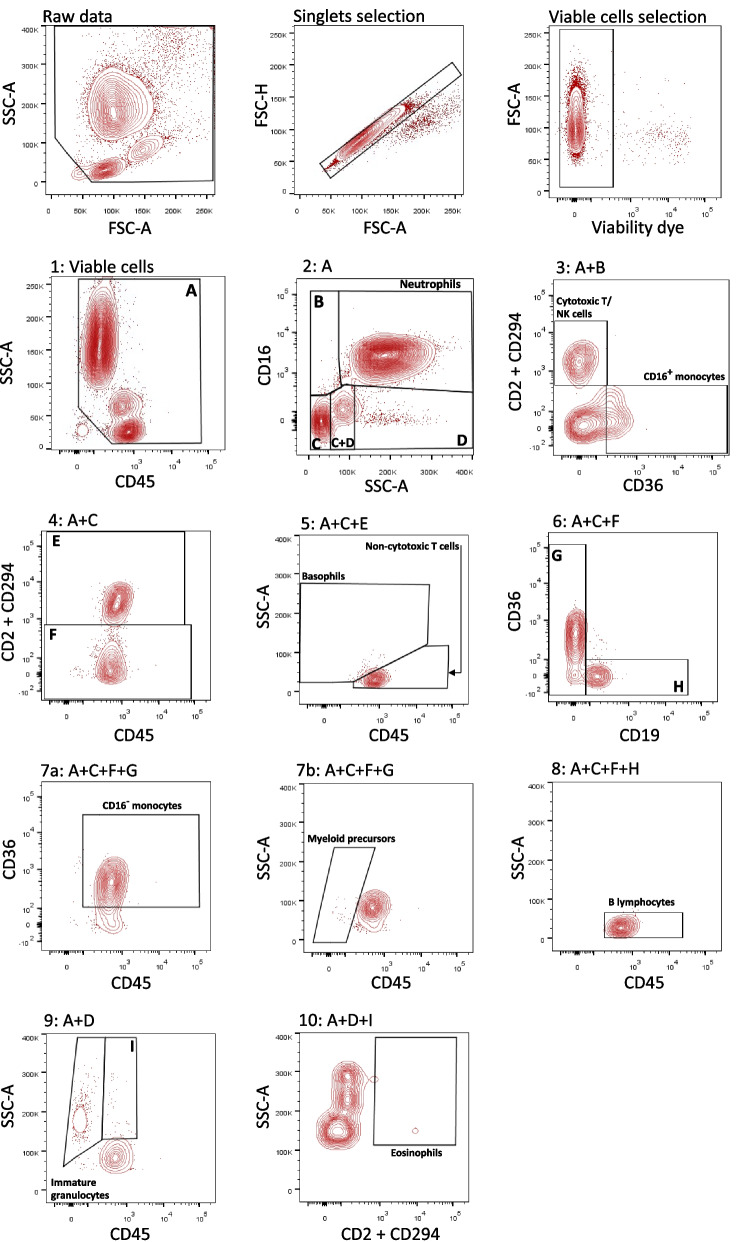
Typical gating strategy applied to peripheral blood to identify 10 leukocyte subpopulations. The data presented were obtained from one representative donor from the lean cohort

**Fig. 2:**
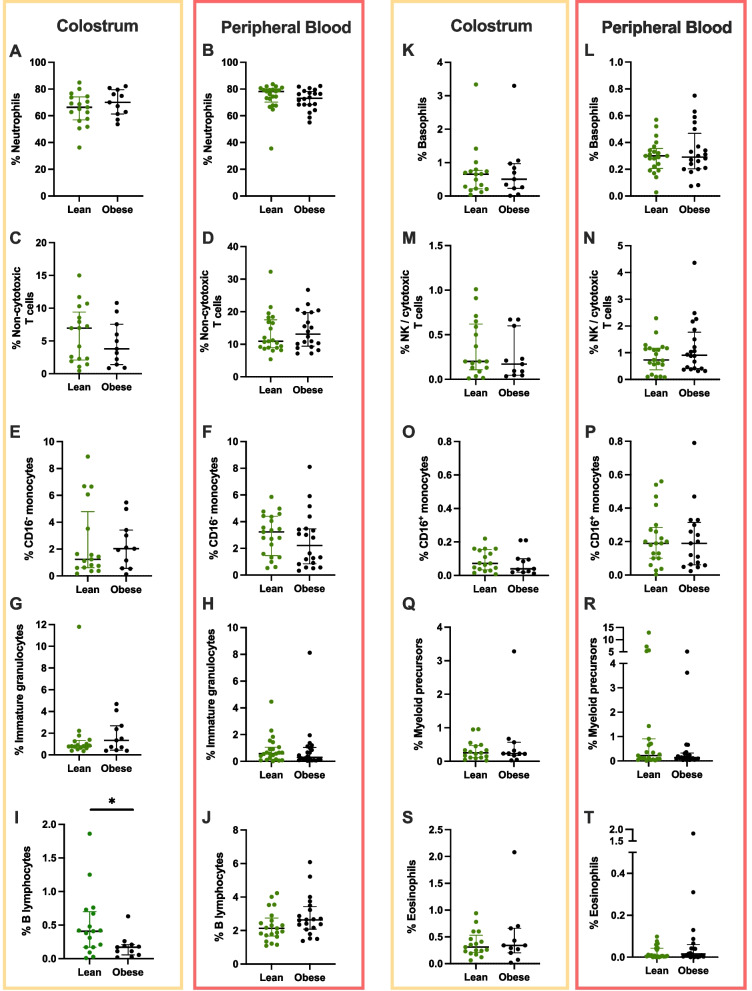
Relative frequencies of leukocyte subpopulations identified in colostrum and peripheral blood of mothers with lean and obese BMI. Individual data points are shown, together with group median and interquartile range, *p*<0.05 (*)

Ten leukocyte subpopulations were confidently identified in peripheral blood using the gating strategy presented in Fig. 1. For all leukocyte proportions measured, there was no difference between the lean and obese cohorts (Fig. 2). Median percentages for each subpopulation, and results of statistical comparisons are presented in Table 3.

**Table 3:** Summary of median leukocyte subset percentages identified in colostrum and peripheral blood of lean and obese cohorts

	**Lean**		**Obese**		**Comparisons **			
	Colostrum	Blood	Colostrum	Blood	Lean	Obese	Colostrum	Blood
**Total leukocytes**	60.30 (43.00-80.00)	96.50 (91.80-98.50)	62 (41.00-87.90)	97.15 (93.70-98.28)	*p*=0.54, *p*=0.80		***p*** **<0.0001,** ***p*** **<0.0001**	
**Neutrophils**	66.40 (56.95-74.10)	78.2 (70.25-80.05)	70 (61.4-79.5)	73.15 (68.25-78.15)	*p*=0.35, *p*=0.48		***p*=0.007**, p=0.51	
**Eosinophils**	0.35 (0.23-0.60)	0.007 (0.004-0.04)	0.34 (0.14-0.67)	0.02 (0.003-0.06)	*p*=0.84, *p*=0.23		***p*<0.0001,** ***p*<0.0001**	
**Basophils**	0.65 (0.22-0.77)	0.3 (0.21-0.36)	0.5 (0.23-0.97)	0.29 (0.20-0.47)	*p*=0.93, *p*=0.46		***p*=0.049**, *p*=0.2	
**Immature granulocytes**	0.81 (0.65-1.26)	0.58 (0.15-1.06)	0.73 (0.42-2.53)	0.30 (0.12-1.04)	*p*=0.90, *p*=0.97		*p*=0.09, ***p*=0.036**	
**Myeloid progenitors**	0.28 (0.11-0.55)	0.22 (0.08-0.91)	0.23 (0.11-0.62)	0.14 (0.08-0.32)	*p*=0.84, *p*=0.17		*p*=0.83, *p*=0.28	
**CD16**^**+**^ **monocytes**	0.07 (0.03-0.16)	0.19 (0.1-0.28)	0.04 (0.02-0.1)	0.15 (0.05-0.31)	*p*=0.49, *p*=0.95		***p*=0.0063**, *p*=0.13	
**CD16**^**-**^ **monocytes**	1.24 (0.62-4.80)	3.23(1.4-4.4)	2.03 (0.57-3.42)	2.2 (0.8-3.4)	*p*=0.78, *p*=0.45		*p*=0.43, *p*=0.83	
**B lymphocytes**	0.41 (0.17-0.70)	2.13 (1.67-2.75)	0.17 (0.06-0.21)	2.63 (2.09-3.43)	***p*=0.029**, *p*=0.09		***p*<0.0001,** ***p*<0.0001**	
**Non-cytotoxic T cells**	6.95 (2.09-9.43)	10.9 (8.94-17.50)	3.81 (1.4-7.56)	13.10 (9.40-19.75)	*p*=0.45, *p*=0.61		***p*<0.0001,** ***p*<0.0001**	
**NK/Cytotoxic T cells**	0.20 (0.11-0.62)	0.73 (0.37-1.17)	0.17 (0.05-0.6)	0.91 (0.41-1.77)	*p*=0.63, *p*=0.17		***p*=0.006,** ***p*<0.0001**	

Frequency of leukocyte subpopulations expressed as median % (interquartile range). Total leukocytes from live singlets are reported and then used as the parent for leukocyte subpopulation frequencies. Non-parametric Mann-Whitney *U* tests were used to compare leukocyte proportions measured per tissue between cohorts (first *p* value = colostrum, second *p* value = blood). Intra-individual comparisons were performed using Wilcoxon matched-pairs signed rank tests, comparing leukocyte proportions between blood and colostrum within each cohort (first *p* value = lean, second *p* value = obese. *p* values < 0.05 were considered significant

Colostrum vs. Blood (p value) within each cohort, analysis btw tissues (Lean, obese)

Obese vs. Lean (p value) (colostrum, blood)

### Obesity is associated with a decreased B lymphocyte fraction in colostrum

**Fig. 3:**
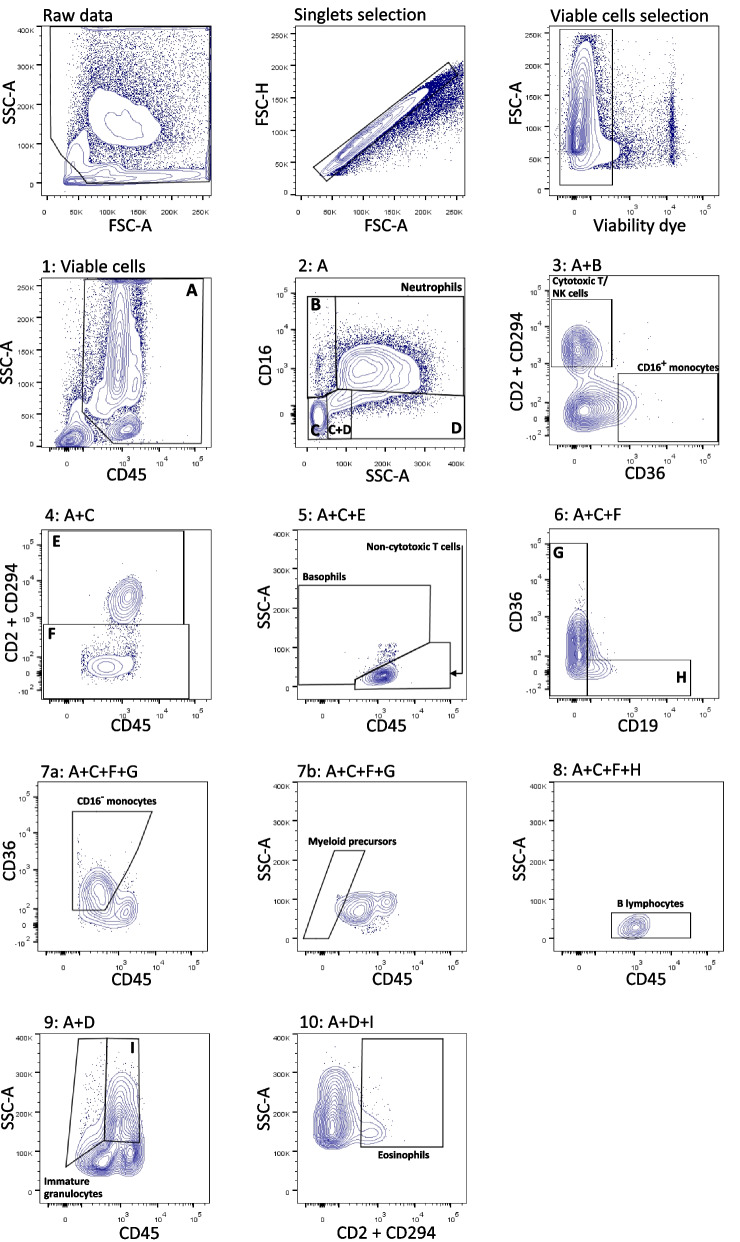
Typical gating strategy applied to colostrum-enriched cells to identify 10 leukocyte subpopulations. Data presented were obtained from one representative donor from the lean cohort

The flow cytometry plots from colostrum samples highlighted consistent differences compared to blood, such as increased debris and doublets proportion, and overall decreased viability (Fig. 3). The B lymphocyte fraction was significantly reduced in the obese cohort compared to the lean cohort (Fig. 2 panel I and Table 3, median 0.17% vs. 0.41% in the lean cohort, *p* = 0.029). The remaining 9 leukocyte subpopulations exhibited similar proportions in colostrum between cohorts. Median % of all leukocyte subtypes for both cohorts are found in Table 3.

### Leukocyte proportions are regulated between blood and colostrum

Neutrophils were the most abundant leukocytes in both tissue types, as expected [31, 35]. There was no difference in neutrophil proportions between groups in either tissue (Fig. 2, Table 3). However, there were significantly less neutrophils in lean mothers’ colostrum compared to lean mothers’ blood, while this difference was not recapitulated in the obese cohort (*p*=0.007 vs. *p*=0.51, respectively, Table 3).

The second highest frequency colostrum leukocytes were non-cytotoxic T cells (medians: 6.95% and 3.81% in lean and obese groups, respectively, Table 3). The frequency of non-cytotoxic T cells in peripheral blood was significantly higher compared to the frequency in colostrum, irrespective of the cohort (Fig. 2, Table 3).

For B lymphocytes, non-cytotoxic T cells, and eosinophils, relative proportions depended on the tissue of origin. Overall, there was a significantly higher fraction of B lymphocytes and non-cytotoxic T cells in peripheral blood compared to colostrum (Fig. 2 I, J and C, D, respectively). On the other hand, there was a significantly higher fraction of eosinophils in colostrum compared to peripheral blood (Fig. 2 S, T). These trends were pervasive across study groups (Table 3). Group-specific differences between tissue were also identified. There were significantly larger fractions of basophils and CD16^+^ monocytes in blood, only in the lean group, and a significantly larger fraction of immature granulocytes in colostrum from the obese group only (Table 3).

### The relative sizes of leukocytes are selectively regulated between blood and colostrum

**Fig. 4:**
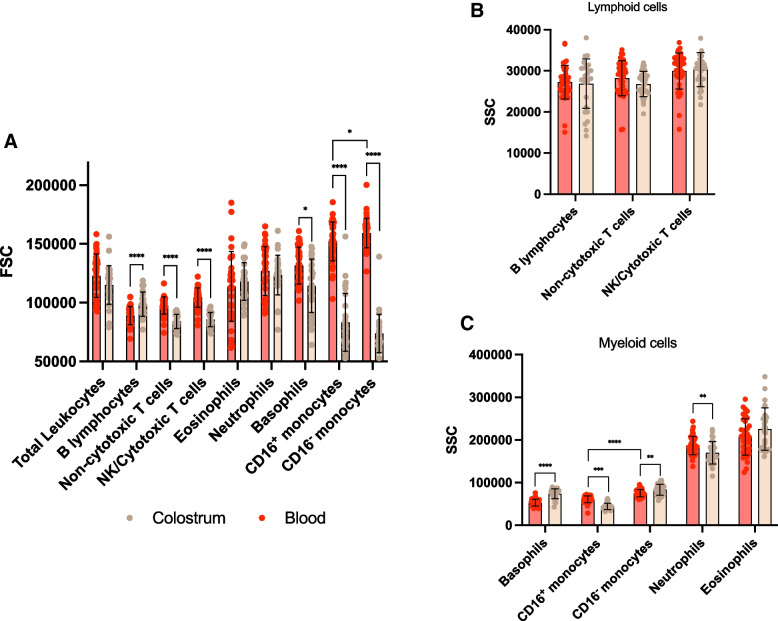
Relative size (FSC) and internal complexity (SSC) of mature leukocytes present in blood and colostrum. Wilcoxon matched-pairs signed rank tests were used to compare leukocyte scattering characteristics in each tissue. The data for each subpopulation pools samples of both lean and obese cohorts, *p*<0.05 (*), *p*<0.01 (**), *p*<0.001 (***), *p*<0.0001 (****)

The relative size of various leukocyte populations varied significantly between tissues as estimated by FSC, and this was pervasive across both cohorts. B lymphocytes were significantly larger in colostrum compared to peripheral blood (Fig. 4A). On the other hand, both populations of T lymphocytes, basophils, and both populations of monocytes were significantly smaller in colostrum compared to blood (Fig. 4A). Of note, in blood, CD16^+^ monocytes were significantly smaller (Fig. 4A, *p* = 0.031) and significantly less internally complex (Fig. 4C, *p* < 0.0001) than classical CD16^−^ monocytes. These results together recapitulate well-described contrasts between these populations, further supporting the identity of these cells. In colostrum, the differences in size and internal complexity between both monocyte subpopulations were exacerbated (*p* < 0.0001 for both). Eosinophils and neutrophils did not exhibit a change in relative size between tissues.

### Leukocytes from the myeloid lineage undergo regulation of internal complexity between blood and colostrum

There was a leukocyte-specific regulation of internal complexity between tissue, as determined by SSC of light, and this finding was pervasive across both cohorts. Lymphoid cells showed stable SSC in blood and colostrum (Fig. 4B), while the SSC of myeloid cells were significantly regulated between tissues (Fig. 4C). Basophils and classical CD16^−^ monocytes exhibited significantly higher SSC in colostrum, while neutrophils and CD16^+^ monocytes exhibited significantly higher SSC in peripheral blood. As seen for FSC properties, eosinophils did not exhibit a change in SSC properties between tissue types.

### Leukocytes regulate CD45 expression between blood and colostrum in a lineage-dependent fashion

**Fig. 5:**
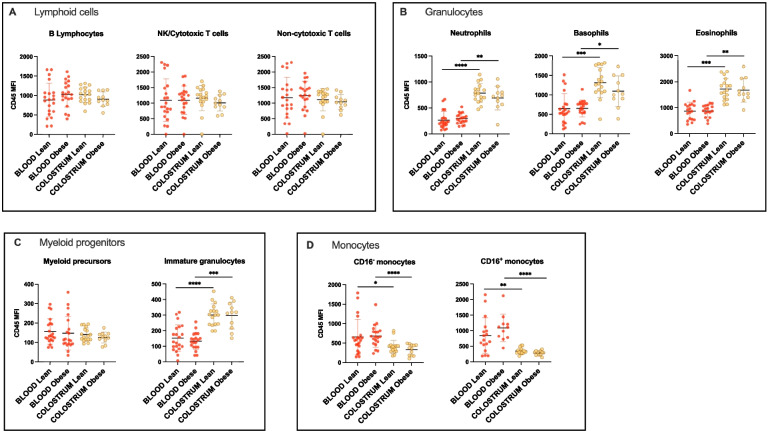
Relative abundance of CD45, expressed as median fluorescence intensity (MFI), on the surface of leukocytes from peripheral blood or colostrum from obese and lean cohorts. Individual values are plotted with mean and SD and Wilcoxon matched-pairs signed rank tests were used to compare intra-individual variations within groups, *p*<0.05 (*), *p*<0.01 (**), *p*<0.001 (***), *p*<0.0001 (****)

Overall, the relative expression of CD45 was higher on lymphocytes, with a mean MFI around 1000 (Fig. 5A) and lower on myeloid progenitors with a mean MFI <400 (Fig. 5C). There was no difference between tissues in the relative expression of CD45 on the surface of cells from the lymphoid lineage (Fig. 5A). All 3 granulocyte subtypes exhibited a significant increase in CD45 expression in colostrum compared to blood (Fig. 5B). Upregulation of CD45 was systematically more significant on granulocytes from the lean cohort. While early myeloid precursors did not exhibit changes in CD45 expression, with MFI consistently averaging around 150 in all groups, colostrum immature granulocytes exhibited twice the levels of CD45 expression observed in peripheral blood, with MFI averaging from 134 and 128 in blood to >300 in colostrum, irrespective of the study groups (Fig. 5C). Both monocyte populations downregulated CD45 expression in colostrum, irrespective of the study group, and the downregulation was systematically more significant in the obese cohort (Fig. 5D).

### Leukocyte-specific regulation of CD16 between peripheral blood and colostrum

**Fig. 6:**
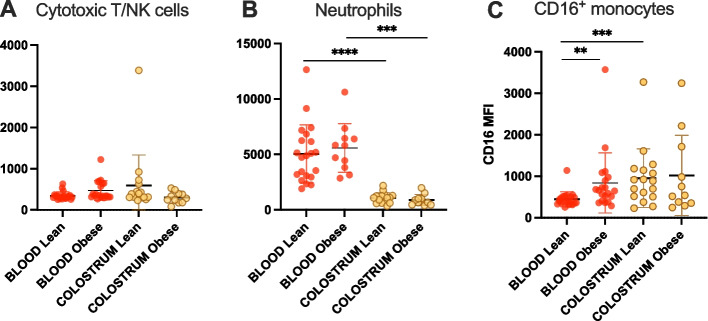
Relative abundance of CD16 expressed as MFI on the surface of relevant leukocyte subpopulations contained in peripheral blood or colostrum samples from obese and lean cohorts. Individual values are plotted with mean and SD, and Wilcoxon matched-pairs signed rank tests were used for intra-individual comparisons, *p*<0.05 (*), *p*<0.01 (**), *p*<0.0001 (****)

We observed stable, relatively low levels of CD16 on cytotoxic T/NK cells across tissues in both study groups (Fig. 6A). Blood neutrophils exhibited the highest expression of CD16 (mean MFI 5049 and 5584 for lean and obese study groups respectively, Fig. 6B) while the expression on blood-circulating monocytes and cytotoxic T/NK cells was overall 10-fold lower. There was a significant downregulation of CD16 on colostrum neutrophils compared to blood, irrespective of the study group, and the difference between tissues was more significant in the lean cohort (Fig. 6B). On the other hand, there was a significant upregulation of CD16 on the surface of colostrum CD16^+^ monocytes, in the lean group only (Fig. 6C). Interestingly, blood-circulating CD16^+^ monocytes expressed significantly more CD16 in the obese cohort than in the lean cohort (*p* = 0.0011, Fig. 6C).

## Discussion

Here, we applied a 7-color panel for flow cytometry to investigate 10 major leukocyte subpopulations in peripheral blood and colostrum from mothers presenting lean or obese BMI [31, 34]. In answering the primary objective of the study, we evidenced a reshaping of the colostrum B lymphocyte compartment in obesity, with less B cells present in the colostrum from mothers suffering from obesity, while all other leukocyte populations remained unaltered in the colostrum between groups. In answering the secondary objective of this study, we identified considerable cell-specific phenotypic alterations of all leukocyte subtypes investigated between blood and colostrum. The alterations evidenced included regulation of cell size, internal complexity, and surface expression CD45 and CD16. Altogether, this report informs for the first time on regulated processes in colostrum leukocytes possibly involved in activation and trafficking from human blood to colostrum and evidences regulations correlated to maternal obesity.

Neutrophil average proportions in colostrum were 1.5 to 5 times higher than previously reported using flow cytometry (medians >65% in both groups, versus less than 15% in [31]), but similar to previously measured in colostrum using a blood hematology analyzer [31, 36]. Blood-circulating neutrophils have a lifespan of a few hours only, which is significantly shorter compared to other leukocytes [37]. Reducing the time between collection and analysis to < 3 h may have allowed increased detection of live neutrophils, compared to longer wait periods in earlier studies. Proportions measured in blood were higher than expected in this tissue, which is consistent with the literature reporting leukocytosis and impaired neutrophil apoptosis during pregnancy and labor [38].

We show that the abundance of CD16 on the surface of neutrophils and of CD16^+^ monocytes is significantly regulated by tissue type, and depending on the cohort. In lean cohort blood, neutrophils express significantly more CD16 while CD16^+^ monocytes express significantly less CD16, than in colostrum. CD16 is a Fc gamma III receptor (FcgIIIR) for the constant fraction of IgG antibodies. CD16 is abundant on the surface of phagocytic cells and its presence correlates with the phagocytic capacity of opsonized pathogens [39]. It is interesting to measure such a regulation for FcR of IgG in colostrum, as the main immunoglobulin isotypes present in colostrum are IgA and IgM, which are not recognized by CD16 [40].

Downregulation of CD16 on colostrum neutrophils could be the result of ectodomain shedding caused by activation or apoptosis. While apoptosis is also generally marked by a decrease in cell size, here no variation was observed in neutrophil relative size between tissue, casting doubt on apoptosis being the cause of CD16 downregulation on neutrophil surfaces in colostrum. Neutrophil activation is a rational alternative in the light of the well-described colostrum microbiota [14, 41, 42]. Finally, CD16 downregulation from colostrum neutrophils may be caused by internalization after cross-linking IgG Fc, in contrast to shedding proposed earlier. Overall, additional experiments are necessary to conclude on the cause of neutrophil CD16 downregulation in colostrum.

Contrasting from findings in neutrophils, in mothers from the lean cohort, the relative abundance of CD16 on CD16-expressing monocytes was higher in colostrum compared to blood. Of note, the present flow cytometry panel was not designed to further subclassify CD16^+^ monocytes between non-classical and intermediate populations, the latter known to express relatively less CD16 than the former.

Therefore, the observed difference could have various origins. There could be an expansion of the higher CD16-expressing non-classical monocytes population, as observed in peripheral blood during infections [43, 44]. A possible alternative could be the upregulation of CD16 from the intermediate population, as previously described [45]. Interestingly, this difference between tissues was not recapitulated in the obese cohort, because CD16 was significantly increased on blood monocytes compared to the lean cohort, to levels that were similar to that of CD16 in colostrum monocytes. This is consistent with obesity involving systemic low-grade inflammation and highlights the relevance of investigating CD16 expression levels on monocytes in addition to other monocyte characteristics known to be modulated by obesity [46]. In the present study, the blood proportion of CD16^+^ monocytes was not perturbated by obesity. It is a possibility that the distinctive post-partum immune profile is causing this discrepancy compared to the obesity-mediated modulations of blood monocytes described in the literature [47]. Overall, it will be necessary to investigate further monocyte subpopulations in colostrum.

CD45 upregulation has been described on granulocytes upon exposure to pathogenic microbes and physiological activators such as fMLP [48–50]. However, the implications of this regulation on the development of the immune response remain unclear, and conflicting results have been described. For example in neutrophils, upregulation of CD45 is consistent with their activation [50]. CD45 is also partially involved in regulating various neutrophil immune functions like cell adhesion, phagocytosis, and ROS production [51]. However, CD45 was also shown to downregulate neutrophil chemotaxis, and in turn, neutrophil ROS production was shown to inhibit CD45 [52, 53]. Therefore, the present results warrant future in-depth analyses of the activation status of granulocytes present in colostrum using functional assays.

Breastmilk is the recommended source of nutrition for infants globally. At present, only exceptional conditions warrant a healthcare professional to consider discouraging this practice, including specific substance abuse but also treatments affecting the immune system of the mother [54–56]. The presented results indicate that suffering from obesity significantly reduces the B lymphocyte compartment in the colostrum, without affecting peripheral blood. Much remains to be investigated about colostrum B lymphocytes in obesity. In peripheral blood, B lymphocytes from obese individuals are more inflammatory and less efficient at switching to memory B cells upon antigen exposure [8]. Here, the features of colostrum B lymphocytes hint toward a phenotype of antibody-secreting cells, with increased cell size, although this remains to be confirmed. Infants born with an immature immune system benefit from the passive transfer of antibodies from their mothers through breastfeeding. This includes immunologically relevant concentrations of immunoglobulins in breastmilk over a long period of time and vaccine-induced antigen-specific IgA and IgG into breastmilk 2-6 weeks post-vaccination [57, 58]. Unvaccinated infants therefore benefit from antibody-mediated protection against infectious diseases, in addition to training of their immature immune system by exposure to these components [59]. Interestingly, a previous study described increased sIgA in obese colostrum [12]. Although more studies are necessary to confirm these findings globally, it is possible that breast-tissue resident plasma cells secrete more sIgA in obesity to compensate for less B cells present in colostrum. The present results therefore suggest obesity may impact the quantity and quality of passive immunity provided to nursing infants.

Additionally, this work provides insights into the regulation of leukocyte trafficking between blood and colostrum since various significant trends were equally recapitulated in both cohorts. Overall, the data indicate minimal regulation of the lymphoid compartment between tissues while myeloid cells were significantly altered morphologically and on the cell surface in colostrum. Mechanisms of leukocyte recruitment to the alveolar lumen during lactation remain largely unknown. Leukocytes are thought to reach breastmilk through the paracellular pathway from a mammary gland origin, crossing tight junctions (TJ), and not by direct extravasation from blood vessels. As TJ are tightly sealed during lactation, it has been suggested that leukocytes are recruited before initiation of lactation [60, 61]. However, a recent study showed increasing numbers of post-mitotic plasma cells in the mouse mammary gland during lactation, suggesting some recruitment may actually take place during lactation [62]. Mouse breastmilk T lymphocytes express TJ proteins, possibly to maintain TJ integrity during leukocyte transmigration during lactation [63]. On the other hand, extravasation is the reported process by which the mammary gland undergoes the initial leukocyte recruitment during pregnancy [64]. In the context of infections, transmigration cause leukocytes to modulate membrane expression of various markers and overall exhibit a proinflammatory profile. This includes enhanced survival for granulocytes and lymphocytes, and increased phagocytosis for neutrophils and monocytes, among other features described in [65]. Transcriptional analysis of the mammary gland throughout gestation, lactation, and weaning showed an upregulation of immune-related function during the involution of the tissue post-weaning, compared to earlier timepoints including lactation [66]. Overall, this suggests a largely unknown complex process physiologically distinct from infection-induced leukocyte transmigration and calls for further investigations into breastmilk leukocyte recruitment.

Early literature has speculated active immunity transfer from breastmilk to neonates [67]. More recently, breastmilk was shown to be significantly enriched in regulatory T cells compared to peripheral blood [68]. This scattered literature implicates a regulation of leukocytes in breastmilk with potential outcomes in the suckling neonate. Here, providing a differential description of leukocyte phenotypes in both tissue types helps to start dissecting this complex and selective recruitment process. We describe that the mothers’ BMI impacts B lymphocyte proportions in colostrum, suggesting a mother’s health status may in turn affect neonatal health.

A possible limitation of this work was relying on BMI to organize cohorts. Various reports demonstrate that BMI alone may not be a sufficient indicator for obesity and % body fat should be used instead [69, 70]. In addition, recruitment and allocation to cohorts were performed during the first trimester of pregnancy, without later revisions of weight gain. We argue that overall first-trimester weight gain has been previously reported as minimal and that mothers suffering from obesity have a lower weight increase compared to lean mothers during this stage of pregnancy [71, 72]. Therefore, the present results may be minimally confounded by differential weight gain during the development of the pregnancy.

Technically, while reporting leukocyte proportions in colostrum provide novel insights, it would be ideal to also measure absolute numbers of cells in colostrum. While earlier work has described absolute counts using BD TruCount tubes, the necessary pre-processing of colostrum samples may challenge the validity of the obtained results. Unfortunately, there is presently no alternative to estimate leukocyte absolute counts in breastmilk, while the physical properties of this tissue hamper their unprocessed use with TruCount tubes. Furthermore, we could not identify all of the leukocytes present in samples, as shown by events outside of population-calling gates, which is nonetheless consistent with the literature [31]. While CD45^+^ leukocytes make up the large majority of nucleated blood-circulating cells, rare CD45^−^ cells such as erythroid precursors or CD45^−^ megakaryocytes were recently reported in healthy individuals which could participate in explaining the < 3.5% CD45^−^ fraction identified in these samples [73, 74].

This report highlights multiple key questions regarding active immunity in human colostrum, that require further study. First, what are the causes of the reduced B cell compartment in obese mothers’ colostrum, and what are the short- and long-term consequences in suckling infants? Why and how are leukocytes trafficked to colostrum, and is the altered phenotype in colostrum a requisite for, or a consequence of trafficking? Finally, the presented data hint toward activation of the innate immune system in colostrum, accentuating the need to investigate colostrum as a complex system, together with its microbiota. Host-microbe crosstalk should be considered in future studies to shed light on the mechanistic regulation of colostrum composition in obesity, and its impact on suckling infants.

## Conclusions

To the best of our knowledge, this is the first study of the main leukocyte subtypes in colostrum from a Latin-American population, the first report of phenotypic alterations of leukocyte subpopulations between peripheral blood and colostrum globally, and the first evidence of obesity altering colostrum leukocytes [25]. Therefore, this pioneering study is a stepping stone to further investigate active immunity in human breastmilk. Additional research is necessary to understand the etiology and consequences of the reported alterations in mothers suffering from obesity.

## Supplementary Information

**Additional file 1: Table S1.** STROBE Statement—checklist of items that should be included in reports of observational studies. **Table S2.** Summary of demographic and clinical parameters of colostrum-blood paired samples study participants. **Table S3.** Comparison of demographic and clinical parameters of study participants in the obese cohort included or excluded from the final analysis.

**Additional file 2: Figure S1.** Flow diagram detailing the successive filters applied to flow cytometry data prior to leukocyte phenotyping.

## Data Availability

The datasets generated and analyzed during the current study are not publicly available due to our manuscript not being published. Raw data (.fcs) are currently available from the corresponding author upon personal request. Upon publication, all datasets and raw data will be available from FlowRepository.com.

## Abbreviations

BMI: Body mass index
FBS: Fetal bovine serum
FcgIIIR: Fragment crystallizable gamma III receptor
fMLP: N-Formylmethionyl-leucyl-phenylalanine
FMO: Fluorescence minus one
FSC: Forward scatter
Ig: Immunoglobulin
IgA: Immunoglobulin A
IgG: Immunoglobulin G
IgM: Immunoglobulin M
MFI: Median fluorescence intensity
PBS: Phosphate-buffered saline
sIgA: Secretory IgA
SSC: Side scatter
TJ: Tight junctions
