# Supplementary material for: A cross-sectional study evidences regulations of leukocytes in the colostrum of mothers with obesity
Source: BMC Med. 2022 Nov 1;20:388. doi: 10.1186/s12916-022-02575-y (PMC9624055; doi:10.1186/s12916-022-02575-y)
Supplement: Supplementary file 1 — Additional file 1: Table S1. STROBE Statement—checklist of items that should be included in reports of observational studies. Table S2. Summary of demographic and clinical parameters of colostrum-blood paired samples study participants. Table S3. Comparison of demographic and clinical parameters of study participants in the obese cohort included or excluded from the final analysis. [file 12916_2022_2575_MOESM1_ESM.docx]

**Table S1.** STROBE Statement—checklist of items that should be included in reports of observational studies.

|  | Item No | Recommendation | Manuscript’s checklist |
| --- | --- | --- | --- |
| **Title and abstract** | 1 | (*a*) Indicate the study’s design with a commonly used term in the title or the abstract. | *Title reads as: “A* ***cross-sectional study*** *identifies […]”.* |
|  |  | (*b*) Provide in the abstract an informative and balanced summary of what was done and what was found. | *Following the recommendations for BMC Medicine, the abstract contains the following successive sections: Background, Methods, Results, Conclusions. All 4 sections contain a comparable amount of information which explains what was done and what was found in a balanced way.* |
| Introduction | | |  |
| Background/rationale | 2 | Explain the scientific background and rationale for the investigation being reported. | *Briefly, here below are successive excerpts from the introduction explaining the rationale for the work presented:*  *-Lines 80-81: “The composition of breastmilk is partially modulated by external parameters such as the mother’s diet, stress levels or health status”*  *-Lines 83-84: “Obesity is […] low-grade systemic inflammation […]”.*  *-Lines 108-109: “Overall maternal obesity is associated with multiple immune-mediated negative outcomes for infants”*  *-Lines 112-113: “Evidence suggest breastmilk-transferred immune factors may impact infant health”*  *-Lines 115-116: “the consequences of obesity on the majority of breastmilk leukocyte populations have not been reported to date”* |
| Objectives | 3 | State specific objectives, including any prespecified hypotheses | *Lines 120-124: “The* ***primary objective*** *of this study was to explore possible variations in leukocyte proportions in the colostrum of mothers suffering from obesity. A* ***secondary objective*** *of this work was to compare proportions and characteristics of leukocytes depending on the tissue of origin: colostrum or peripheral blood.”* |
| Methods | | |  |
| Study design | 4 | Present key elements of study design early in the paper. | *Line 159: “****We conducted a cross-sectional study*** *of leukocyte subpopulations in blood and colostrum of mothers with BMI <25 and BMI >30.”. Participants, place and time of recruitment and sampling, amongst additional relevant data, are described* |
| Setting | 5 | Describe the setting, locations, and relevant dates, including periods of recruitment, exposure, follow-up, and data collection. | *All relevant information is included in the subsection “Study design and participants” starting line 158 of the revised manuscript. Briefly,*  ***Setting****: Line 165: “Hospital Regional Materno Infantil”*  ***Location****: Line 166: “Nuevo León, Mexico”*  ***Relevant dates****: 1-****Period of recruitment:*** *February 2020-August 2020*  *(implied, from line 166-168: “adult mothers were invited to participate in the study during the first obstetric consultation occurring during the first trimester of gestation”). 2-****Data (sample) collection****: line 165: “between October 2020 and March 2021”.*  ***Exposure****: Lines 200-203 “Oxytocin was not used during the first stages of labor. However, oxytocin was prescribed in 34 of 41 subjects (84%), during the first 8 hours after delivery, as per international recommendations.”*  ***Follow-up*** *information is not available (not part of the study design).* |
| Participants | 6 | (*a*) *Cohort study*—Give the eligibility criteria, and the sources and methods of selection of participants. Describe methods of follow-up  *Case-control study*—Give the eligibility criteria, and the sources and methods of case ascertainment and control selection. Give the rationale for the choice of cases and controls  ***Cross-sectional study***—Give the eligibility criteria, and the sources and methods of selection of participants. | ***Selection criteria:*** *Lines 187-198: “****Eligibility*** *to participate in the study was determined on the basis of: 1) mother’s age between 18 and 34 years, 2) adequate prenatal visits without any adverse event during pregnancy 3) pre-pregnancy BMI <25 or >30, 4) term infant, and 5) willingness to participate.* ***Exclusion criteria*** *included: 1) having received antibiotics anytime during the 3-month period before birth, or having received a prolonged antibiotic treatment (>3 months) anytime during pregnancy, 2) having received immunosuppressive doses of steroids during pregnancy, 3) previous monoclonal antibody treatment, 4) history of chronic disease (outside of obesity), 5) suffering from any dietary disease, 6) episodes of diarrhea during the last 2 weeks of pregnancy, 7) history of surgery within 12 months prior to pregnancy, 8) history of antineoplastic treatment.* ***Elimination criteria*** *included: 1) having received antibiotics for >24 h post-birth, 2) necessity of ICU care of the neonate 3) any additional cause impeding sample collection.*  ***Source and methods of selection of participants:*** *Lines 166-167: “adult mothers […] were invited to participate in the study during the first obstetric consultation occurring during the first trimester of gestation.” Signed informed consent was obtained, as per line 164*. |
|  |  | (*b*) *Cohort study*—For matched studies, give matching criteria and number of exposed and unexposed  *Case-control study*—For matched studies, give matching criteria and the number of controls per case | *Not applicable* |
| Variables | 7 | Clearly define all outcomes, exposures, predictors, potential confounders, and effect modifiers. Give diagnostic criteria, if applicable. | ***Independent and dependant variables:*** *Lines 196-201: “Regarding the variables of the study, the main independent variable and* ***hypothesized outcome predictor*** *was the BMI, calculated from self-reported pre-pregnancy weight and size. Additional variables collected or measured in this work included participant’s age, primiparity (yes/no), infant gender, gestational age at birth, type of delivery (vaginal/C-section), weight of infant at birth, volume of colostrum obtained, frequency of leukocyte subpopulations in blood and colostrum samples.”*  ***Exposure:*** *Lines 200-203: “Oxytocin was not used during the first stages of labour. However, oxytocin was administered in 34 of 41 subjects (84%), during the first 8 hours after delivery, as per international recommendations”.*  ***Diagnostic criteria:*** *lines 185-187 “Participants were allocated to the obese cohort (BMI >30) or lean cohort (BMI <25), according to declared pre-pregnancy weight during the first visit, and in accordance with the WHO classification guidelines[1].”* |
| Data sources/ measurement | 8* | For each variable of interest, give sources of data and details of methods of assessment (measurement). Describe comparability of assessment methods if there is more than one group. | *-****BMI:*** *calculated as from self-reported pre-pregnancy weight (kg) divided by measured size squared (m^2^).*  *-****Participant’s age****, was self-reported during prenatal visit*  *-****Primiparity*** *(yes/no), was self-reported during prenatal visit*  *-****Infant gender****, was defined at birth by attending pediatrician*  *-****Gestational age at birth****, was defined at birth by attending obstetrician/gynecologist, according to calculation based on date of last menstrual period, or, when unavailable, estimated from the somato-neurologic characteristics of the neonate.*  *-****Type of delivery*** *(vaginal/C-section), was noted on day of birth by medical team*  *-****Weight of infant at birth****, was measured at birth by attending paediatrician*  *-****Volume of colostrum collected****, was measured at the start of sample processing by scientist*  *-****Frequencies of leukocyte subpopulations in blood and colostrum*** *samples were calculated from flow cytometry analyses.* |
| Bias | 9 | Describe any efforts to address potential sources of bias | *The clinical and demographic parameters of patients were compared in both study groups independently for blood (Table 1) and for colostrum (Table 2), as analyses could not be tissue (blood-colostrum) matched, which could have introduced bias in result analyses..* |
| Study size | 10 | Explain how the study size was arrived at | *We considered qualitative factors such as the sample size of previous similar studies (for example: 15 samples per group from study [2], 8 samples per group from [3], 25 test samples from [4], 10 samples per group in [5]), and resource constraints in determining study size. To the best of our knowledge this is the first report of colostrum leukocyte analyses in obese mothers, therefore no a priori sample size calculation could be performed.* |
| Quantitative variables | 11 | Explain how quantitative variables were handled in the analyses. If applicable, describe which groupings were chosen and why | *Methods for handling quantitative variables were described in the Methods section* |
| Statistical methods | 12 | (*a*) Describe all statistical methods, including those used to control for confounding | *All statistical methods were described in the Methods section* |
|  |  | (*b*) Describe any methods used to examine subgroups and interactions | *All statistical methods were described in the Methods section* |
|  |  | (*c*) Explain how missing data were addressed | *Suboptimal samples were removed from analysis as per item 9 and described in the Methods section. There was no other case of missing data.* |
|  |  | (*d*) *Cohort study*—If applicable, explain how loss to follow-up was addressed  *Case-control study*—If applicable, explain how matching of cases and controls was addressed  *Cross-sectional study*—If applicable, describe analytical methods taking account of sampling strategy | *Subject´s inclusion to the study was done on a sequential, non-probabilistic basis, on those mothers admitted to the study hospital in order to receive medical attention during delivery* |
|  |  | (*e*) Describe any sensitivity analyses | *Sensitivity analyses were not performed, per study design* |

| Results | | | Manuscript’s checklist |
| --- | --- | --- | --- |
| Participants | 13* | (a) Report numbers of individuals at each stage of study—eg numbers potentially eligible, examined for eligibility, confirmed eligible, included in the study, completing follow-up, and analysed | *The number of participants is reported, there was no follow-up (cross-sectional study).* |
|  |  | (b) Give reasons for non-participation at each stage | *Not applicable* |
|  |  | (c) Consider use of a flow diagram |  |
| Descriptive data | 14* | (a) Give characteristics of study participants (eg demographic, clinical, social) and information on exposures and potential confounders | *Participants clinical and demographic characteristics are reported and analysed* |
|  |  | (b) Indicate number of participants with missing data for each variable of interest | *Number of eliminated colostrum samples is reported* |
|  |  | (c) *Cohort study*—Summarise follow-up time (eg, average and total amount) | *Not applicable* |
| Outcome data | 15* | *Cohort study*—Report numbers of outcome events or summary measures over time | *Not applicable* |
|  |  | *Case-control study—*Report numbers in each exposure category, or summary measures of exposure | *Not applicable* |
|  |  | *Cross-sectional study—*Report numbers of outcome events or summary measures | *Numbers are reported* |
| Main results | 16 | (*a*) Give unadjusted estimates and, if applicable, confounder-adjusted estimates and their precision (eg, 95% confidence interval). Make clear which confounders were adjusted for and why they were included | *Proportions of leukocyte subpopulations, expressed as median (interquartile range). No confounders were identified.* |
|  |  | (*b*) Report category boundaries when continuous variables were categorized |  |
|  |  | (*c*) If relevant, consider translating estimates of relative risk into absolute risk for a meaningful time period | *Not applicable* |
| Other analyses | 17 | Report other analyses done—eg analyses of subgroups and interactions, and sensitivity analyses | *All analyses performed were reported in Methods* |
| Discussion | | |  |
| Key results | 18 | Summarise key results with reference to study objectives | *Key results with reference to the study objectives are summarized in the first paragraph of the discussion.* |
| Limitations | 19 | Discuss limitations of the study, taking into account sources of potential bias or imprecision. Discuss both direction and magnitude of any potential bias |  |
| Interpretation | 20 | Give a cautious overall interpretation of results considering objectives, limitations, multiplicity of analyses, results from similar studies, and other relevant evidence | *The interpretation of the results was very cautious, given the complete novelty of the investigation, cross-sectional nature of the study and relatively low sample size which all together do not allow to infer causality.* |
| Generalisability | 21 | Discuss the generalisability (external validity) of the study results |  |
| Other information | | |  |
| Funding | 22 | Give the source of funding and the role of the funders for the present study and, if applicable, for the original study on which the present article is based | *Funders are acknowledged in the relevant manuscript section.* |

*Give information separately for cases and controls in case-control studies and, if applicable, for exposed and unexposed groups in cohort and cross-sectional studies.

**Note:** An Explanation and Elaboration article discusses each checklist item and gives methodological background and published examples of transparent reporting. The STROBE checklist is best used in conjunction with this article (freely available on the Web sites of PLoS Medicine at http://www.plosmedicine.org/, Annals of Internal Medicine at http://www.annals.org/, and Epidemiology at http://www.epidem.com/). Information on the STROBE Initiative is available at [www.strobe-statement.org](http://www.strobe-statement.org).

**Table S2.** Summary of demographic and clinical parameters of colostrum-blood paired samples study participants

|  | **Cohort** | | ***p*** |
| --- | --- | --- | --- |
| **Variable** | **Lean** | **Obese** |  |
| N total | 17 | 11 |  |
| Maternal age (years), median (IQR) | 23 (22-26) | 26 (18-29) | 0.82 |
| Maternal BMI (kg/m^2^), median (IQR) | 22.8 (20.6-24.1) | 34.9 (33.6-35.8) | **<0.001** |
| Primiparous, N (%) | 4 (24) | 5 (45) | 0.23 |
| Infant gender, N females (% total) | 8 (47) | 7 (64) | 1 |
| Gestational age (weeks), median (IQR) | 39 (38-40) | 39 (38-40) | 0.09 |
| Delivery type, N V (%) | 13 (71) | 4 (36) | 0.73 |
| Infant birth weight (g), median (IQR) | 3320 (2915-3842) | 3425 (3220-3670) | 0.46 |
| Volume of colostrum obtained (mL), median (IQR) | 2.5 (2.0-3.0) | 1.5 (1.0-1.8) | 0.12 |

N: Number of events, BMI: Body Mass Index, V: vaginal births, IQR: Interquartile Range.

Statistically significant p values for calculated differences are depicted in bold type. Continuous data were analyzed with Mann-Whitney’s U test, proportions were compared with the Fisher´s exact test.

**Table S3.** Comparison of demographic and clinical parameters of study participants in the obese cohort included or excluded from the final analysis.

|  | **Obese cohort, n =20** | | ***p*** |
| --- | --- | --- | --- |
| **Variable** | **Included** | **Excluded** |  |
| N total = 20 | 11 | 9 |  |
| Maternal age (years), median (IQR) | 26 (18 – 29) | 29 (22 – 29) | 0.50 |
| Maternal BMI (kg/m^2^), median (IQR) | 34.5 (33.6 – 35.8) | 34.4 (33.6 – 35.0) | 0.41 |
| Primiparous, N (%) | 5 (45.5) | 1 (11.1) | 0.10 |
| Infant gender, N females (% total) | 4 (36.4) | 4 (44.4) | 0.33 |
| Gestational age (weeks), median (IQR) | 39 (38 – 40) | 39 (38 -39.5) | 0.50 |
| Delivery type, N V (%) | 7 (63.6) | 6 (66.7) | 0.36 |
| Infant birth weight (g), median (IQR) | 3425 (3320 – 3670) | 3400 2968 – 3588) | 1.0 |
| Volume of colostrum obtained (mL), median (IQR) | 1.5 (1.0 – 1.8) | 1.5 (1.0 -1.85) | 1.0 |

N: Number of events, BMI: Body Mass Index, V: vaginal births, IQR: Interquartile Range.

Statistically significant p values for calculated differences are depicted in bold type. Continuous data were analyzed with Mann-Whitney’s U test, proportions were compared with the Fisher´s exact test.
