# Supplementary material for: A cross-sectional study evidences regulations of leukocytes in the colostrum of mothers with obesity
Source: BMC Med. 2022 Nov 1;20:388. doi: 10.1186/s12916-022-02575-y (PMC9624055; doi:10.1186/s12916-022-02575-y)
Supplement: Supplementary file 2 — Additional file 2: Figure S1. Flow diagram detailing the successive filters applied to flow cytometry data prior to leukocyte phenotyping. [file 12916_2022_2575_MOESM2_ESM.pptx]

## Slide 1
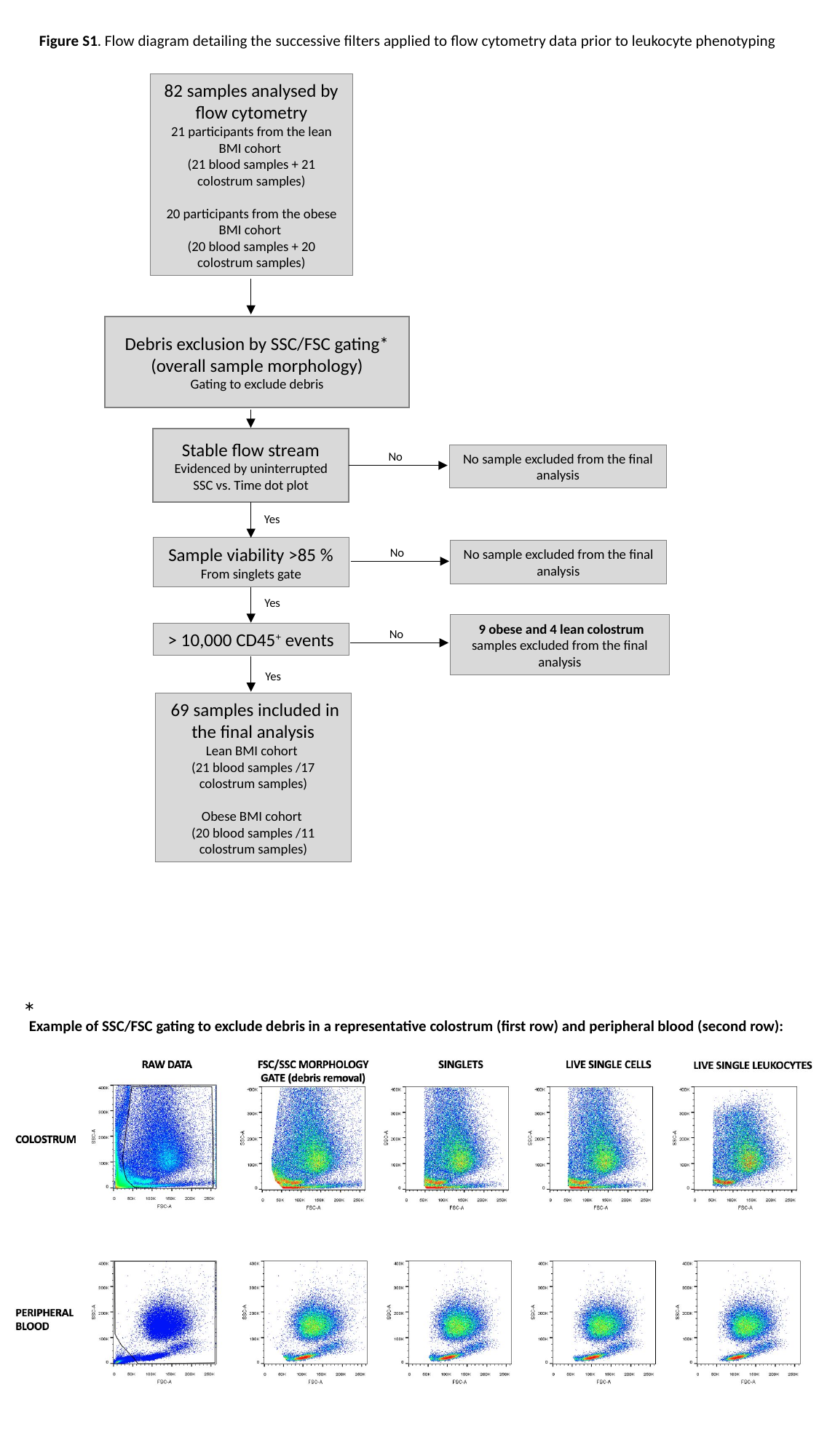

Figure S1. Flow diagram detailing the successive filters applied to flow cytometry data prior to leukocyte phenotyping
82 samples analysed by flow cytometry
21 participants from the lean BMI cohort
(21 blood samples + 21 colostrum samples)
20 participants from the obese BMI cohort
(20 blood samples + 20 colostrum samples)
Debris exclusion by SSC/FSC gating* (overall sample morphology)
Gating to exclude debris
Stable flow stream Evidenced by uninterrupted SSC vs. Time dot plot
No
No sample excluded from the final analysis
Yes
Sample viability >85 %
From singlets gate
No
No sample excluded from the final analysis
Yes
 9 obese and 4 lean colostrum samples excluded from the final analysis
No
> 10,000 CD45+ events
Yes
 69 samples included in the final analysis
Lean BMI cohort
(21 blood samples /17 colostrum samples)
Obese BMI cohort
(20 blood samples /11 colostrum samples)
*
Example of SSC/FSC gating to exclude debris in a representative colostrum (first row) and peripheral blood (second row):
